# Renovation priorities for old residential districts based on resident satisfaction: An application of asymmetric impact-performance analysis in Xi’an, China

**DOI:** 10.1371/journal.pone.0254372

**Published:** 2021-07-22

**Authors:** Yijun Liu, Huimin Li, Wenlong Li, Sunmeng Wang

**Affiliations:** 1 School of Civil Engineering, Xi’an University of Architecture and Technology, Xi’an, China; 2 School of Management, Xi’an University of Architecture and Technology, Xi’an, China; Northeastern University (Shenyang China), CHINA

## Abstract

China is currently designing a regional economic layout for high-quality urban development, shifting its focus from the primary stage of beautifying cities to the next stage of profound urbanization aiming at strengthening industry. This is of high importance given that the urbanization rate of permanent residents by the end of 2019 was 60.60%, according to the National Bureau of Statistics of China. In the face of various factors such as the need to economize the intensive use of resources, urban stock development, and the need for urban constructions to maintain harmony with the surrounding ecosystem, regeneration has emerged as an efficient means to repurpose old residential districts. It conforms to the policy of stock planning and is one of the important methods to ensure the sustainable development of a city. Prioritizing the right attributes in renovation is one of the critical steps in the regeneration of old residential districts—instead of merely focusing on their selection and scoring by experts, more attention should be paid to the resident satisfaction (or the lack thereof) arising from them. Therefore, in this study, we have proposed a collaborative approach that requires communities to prioritize the appropriate aspects in urban renewal. This study employed the three-factor theory of customer satisfaction, to investigate the five attributes namely geographical location, infrastructure, traffic, residential management, and living facilities, of satisfaction and dissatisfaction of residents in the old city. This includes 327 samples based on the residents of Yanta old town in Xi’an. The asymmetric impact-performance analysis technique was used to explore, and quantify the asymmetric relationship between the attributes of old residential districts, and residents’ satisfaction. The results proved that attributes were divided into three: excitement factors, performance, and basic factors, based on the asymmetric influence of attributes on residents’ satisfaction. Residential management was into excitement factors, living facilities were categorized into basic factors, and the remaining three attributes were categorized into performance factors. The satisfaction of the residents regarding the renovation was maximized by comprehensively considering the performance of given attributes, adjusting the improvement strategies of each attribute, and further determining the focus of the renovation of the old residential districts. Simultaneously, it helped planners make more rational choices in urban renewal and sustainable development.

## 1. Introduction

**Fig 1:**
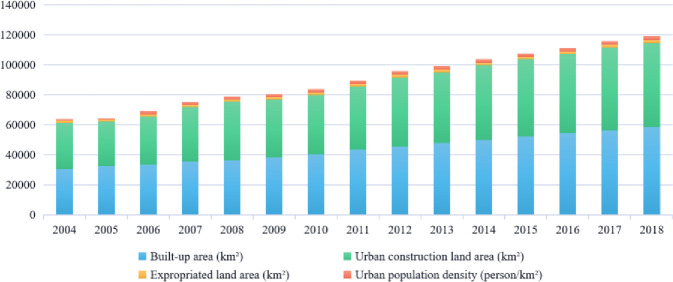
Changes in urban construction from 2004 to 2018.

The rapid development of Chinese society and economy in recent years has accelerated urbanization, simultaneously opening up opportunities for and placing tremendous pressure on city construction. The extensive development mode of high investment, consumption, and pollution has also led to environmental pollution and ecological damage, which has affected the health, and hampered the sustainable development status of the city. Land resources per capita are extremely scarce [1]. However, the rate of land urbanization is much faster than the rate of population urbanization. From 2004 to 2018, the national urban construction land area increased from 30,781.28 (km^2^) to 5,6075.9 (km^2^), and the urban population density ranged from 865 (person/km^2^) to 2546.17 (person/km^2^), which is also shown in Fig 1. A large amount of stock space in the city is being used inefficiently. With the acceleration of urbanization [2], the development of urban construction is gradually shifting from external expansion to connotation improvement. It has transitioned from large-scale incremental construction to a new stage, where both inventory renewal and incremental adjustment are equally important. As a result, a large number of restoration projects such as industrial parks, historical and cultural blocks, old residential districts, and shanty towns, continue to be introduced.

**Fig 2:**
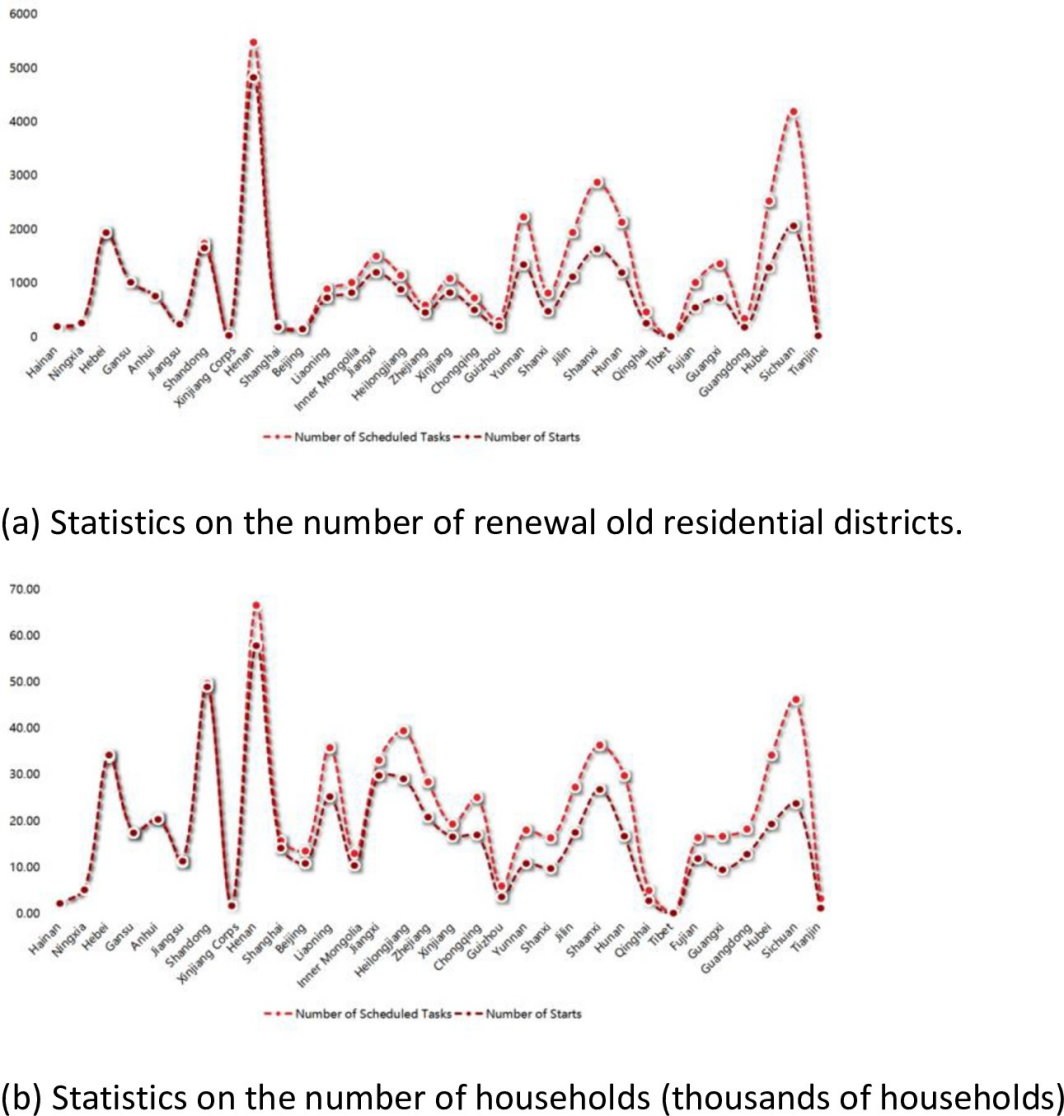
The progress of renovating old residential districts. (a) Statistics on the number of renewal old residential districts. (b) Statistics on the number of households (thousands of households).

In China, the construction and development of old residential districts are mainly concentrated in three time periods, that is, from the early days of the founding of the People’s Republic of China to the 1980s, from the 1980s to 2000, and from 2000 to the present. Among these, the residential areas in the first phase fulfilled their lives and have been demolished and rebuilt on a larger scale, due to severe physical aging, and poor functionality of the initial planning and design of the construction. Since, the old residential districts from 2000 to the present, had a relatively late construction time, the planning and construction of housing and supporting facilities, are near completion. Additionally, the physical aging phenomenon was not obvious, hence, it was not included in the scope of the old residential district renovation objects. This research mainly refers to the old residential areas that were built in the 1980s and 1990s, and are still in use. Due to the age of construction, low construction standards and improper maintenance, these areas do not meet the needs of a modern life for the residents. Therefore, it is an urgent requirement to renovate existing urban settlements [3,4]. At the national level, in order to resolve a series of problems in old residential areas, the government actively promotes the renovation of old residential districts (RORD), and has issued various relevant policies to guide the renovation process. In 2016, in the "Several Opinions of the State Council on Further Strengthening the Management of Urban Planning and Construction", it was proposed that "the orderly implementation of urban repairs and organic renewal to solve the problems of deterioration of the environmental quality of the old city, the chaos of spatial order, and the destruction of historical and cultural heritage". The old residential district renovation is not only able to improve the living environment of residents, but also a necessary content for the renewal of the old urban area, and a necessary factor to promote urban renewal and development. In 2018, the government work report emphasized "the orderly implementation of the renovation of old communities, the improvement of supporting facilities, and the encouragement of conditional installation of elevators" [5]. In 2019, it was clearly pointed out that the renovation of old communities is a shortcoming of the city. In 2020, a document issued by the General Office of the State Council proposed vigorous renovation and upgradation of old communities in cities, improvement in the living conditions of residents, and contribution to convenience, comfort and betterment in people’s lives [6]. Subsequently, the National Development and Reform Commission pointed out that it will comprehensively promote the renovation of old communities in cities, improve the quality of living, and promote the implementation of urban renewal actions. Alongside urban renewal, the concept of "resilience" has also been integrated into the renovation of old residential areas, and leaks and vacancies have been fully checked. China is accelerating the improvement of the water, electricity, gas, road, pipe network and other systems of old communities, constantly improving functions and structures, improving the ability of urban old residential areas to resist various disaster risks, and striving to build safer and more resilient cities. As of December 2018, 106 such projects have been completed [7]. According to MOHURD, implementation of an additional 39,000 renovation projects, impacting nearly 7 million households in old residential districts in various parts of China, was planned for the year 2020; this is double the number of projects completed in 2019 [8]. Fig 2 shows the progress in renovation of old residential districts, till August 2020.

Renewal is the eternal theme of the city, and the source of the its vitality [9]. For example, Ju’er Hutong, a pilot project constructed for the preservation of the Beijing old city, retained its original construction in the renovation. It integrated multi-story residential buildings with traditional courtyards, to conserve the prevailing street and architectural culture. It is also in line with the direction of development of the city. Therefore, many scholars have explored the renewal of old residential areas from different perspectives. Some scholars have studied the renewal from a macro level of urban renewal and development [10]. The vulnerability of society and cities, and the heat island effect has increased, due to the continued global warming trend [11]. Sponge city construction has become one of the preferable methods to solve urban problems [12]. Liu [13] proposed a regeneration model in Wuhu using sponge-city technology, and Zhai et al. [14] studied the analysis and application of sponge-city construction technology to the design of old residential districts. He B. et al. [15] chose China’s low-carbon ecological cities, green universities, and green buildings as representatives for cities, communities, and building scales, and detailed their connection. Gu T. et al. [16] studied the satisfaction of community residents regarding the transformation of the sponge-style old community. Wang [17] studied the design aspects of public transport retrofitting in old residential districts, while Danni Ke [18] evaluated the benefits of applying sponge-city technology to these districts. At the micro level, from the planning and design perspective, Cheng [19] proposed a design strategy for multiple stakeholders such as designers, governments, and residents to convene and work together to improve the environmental quality of old residential districts. Chen J. et al. [20] put forward the concept of community micro-reconstruction, with an aim to improve the daily life of residents, based on the public space and public service facilities of the old community, without adjusting the planning indicators.

From an energy-saving transformation perspective, Ouyang J. et al. [21] analyzed the economic benefits of the energy-saving renovation measures, through the life cycle cost (LCC) method. Huang J. et al. [22] studied the thermal performance optimization of the envelope structure in the energy-saving renovation of existing houses. Weinsziehr T. et al. [23] analyzed the relationship between buildings with a high potential for final energy reduction, and a concentration of low-income, older or empty-nest households. In order to achieve a low-carbon and just society, possible actions are suggested, for buildings with elevated barriers, for energy renovation in shrinking cities. From the perspective of social cognition, Guo B. et al. [24] established a cognitive theoretical model of owners’ participation in community management based on a framework of interaction among individuals, environment and behavior. This study explains the reason behind the failure of the government’s urban renewal policy in resolving the old residential management difficulties. Furthermore, we have provided solutions for establishing a long-term development model, which involves active owner participation, by redefining the laws, and disclosing the old communities’ planning to alter their pessimistic expectations. Duan X. et al. [25] constructed a public participation model, and formulated a public participation guarantee system to enable active participation by the public, in the renewal of old communities. Through in-depth participation to provide residents with a voice channel, in order to meet their desire to participate in community management, we can create an inclusive and shared community environment, and promote grassroots social governance. From the perspective of case study, Fan X. et al. [26] used the example of 50 communities in Dalian to conduct an empirical analysis. The results showed that the transforming needs of old communities include accessibility, building and housing, community care and activities, and spiritual needs. Shukurov I.S et al. [27] established a mathematical model and used it to determine the best renovation plan for the old residential area. The proposed methodology makes it possible to eliminate the influence of possible subjectivity of expert judgments. It instead, withdraws from the expert method of optimality appraisal of various options of existing municipal development reconstruction. M. Reza Shirazi et al. [28] proposed a tripartite framework for measuring social sustainability of urban neighborhoods, which combines the three elements of neighborhood, neighboring, and neighbors. Further, they applied it to Bethnal Green in London, and put forward practical suggestions to promote the sustainable development of the Bethnal Green society.

It can be observed that previous studies mainly included macro and micro levels. However, scholars have explored the recycling of old buildings, and revitalized the existing land from the perspectives of urban renewal, resilient urban construction, and sustainable urban development. The micro level includes urban planning and architectural design, green buildings and energy-saving renovations, management of old communities, and empirical research on special cases. Most of the existing research focuses on the old residential districts as the research object, starting from the old community itself, to discuss the key points of reconstruction and upgrading strategies. This often overlooks the feelings of the residents in the settlement. Residents are the direct beneficiaries of the RORD. They have a large age span, and almost cover the different needs of residential environment transformation at all ages. Therefore, it is necessary to give priority to, and meet the requirements and expectations of residents. In other words, the community should be involved in discussion and education; and the living conditions and environment of the old community should be better transformed with actual needs and technical means. Additionally, the renewal and transformation of the old community should be promoted, and the existing land must be used to contribute to the sustainable development of the city.

Using data from residents in the old city of Xi’an, China, combined with the three-factor theory [29–33], this study uses asymmetric impact performance analysis (AIPA) technology to determine the focus of the RORD. According to the potential asymmetric influence of the attributes on resident satisfaction, they are classified into three categories—basic factors, excitement factors, and performance factors. By considering the performance of these attributes, those that need to be prioritized during RORD were further determined.

## 2. Methods

### 2.1 Residents’ satisfaction as an indicator of the priority of RORD

#### 2.1.1 The origin of the concept of resident satisfaction

RORD projects span a wide range of activities and involve many residents; therefore, the scope of renovation must be made clear. At the same time, the actual results of renovating a community are reflected in the impact felt by its residents. Hence, this paper analyzes resident satisfaction and uses it to suggest modifications to the methods used in RORD in order to better meet the needs of the residents [34]. Resident satisfaction is an expression of public satisfaction, which can be measured by a community satisfaction scale. Public satisfaction was first proposed by Osborne and Gaebler, who believed that the concept of customer satisfaction was applicable to governance as well [35]. Public satisfaction is a quantitative description of customer satisfaction [36]. Customer satisfaction is a difference function that compares the actual value derived by a customer with the expected value, while public satisfaction is a concept where, "in the process of experiencing public administration, the public’s needs are met, and the public’s expectations are consistent with actual feelings, so that the public has a positive attitude of affirmation, joy, and satisfaction [37]." Resident satisfaction, quantified by the difference between actual and expected living environments, is simply the satisfaction experienced by residents living in a specific place.

#### 2.1.2 Determine the priority of influencing factors based on residents’ satisfaction

Research on this topic primarily focuses on evaluating the community performance index, which is a key indicator of resident satisfaction [32]. Davis et al. [38] considered public transport to be a main factor influencing community resident satisfaction, whereas Hur et al. [39] emphasized on building density and vegetation rates as the key elements. Lovejoy et al. [40] used factor analysis and a logit model to find that neighborhood safety and appearance, along with resident vitality and diversity, significantly impact resident satisfaction in suburban and urban areas. Li [41] conducted a survey of migrants residing in Beijing, Shanghai, and Guangzhou, and found social belonging to have the highest impact on their residential satisfaction. Lee et al. [42] studied the impact of objective characteristics of a community and the subjective feelings of residents on their satisfaction. Li et al. [43] considered residents’ satisfaction as the starting point to evaluate community housing, public facilities, land rights, employment, social security, and community environment built in an eco-friendly manner in the city of Suzhou. Guo [44] used factor analysis to study four aspects influencing resident satisfaction in urban village reconstruction: benefit distribution, program disclosure, social transformation structure, and historical culture, while Zhu [45] used four other features—life, management, service, and emotion—to study the relationship between community informatization and resident satisfaction.

Previous studies have indicated the presence of several factors influencing resident satisfaction. These factors are classified into two categories: the objective environmental characteristics of the districts and the subjective perception of the residents [29,46,47]. The Campbell model [48] pointed out that resident satisfaction arises from the subjective emotions stimulated by the objective environment—residents cognitively experience stimuli from their living environment and make subjective evaluations of it based on their individual criteria, which then shapes their satisfaction [49]. Therefore, determining the dominant factors and analyzing the primary and secondary relationships between them to ascertain which factors need to be prioritized during renovation are pressing scientific problems that need to be solved using RORD.

### 2.2 The three-factor theory and asymmetric impact-performance analysis

**Fig 3:**
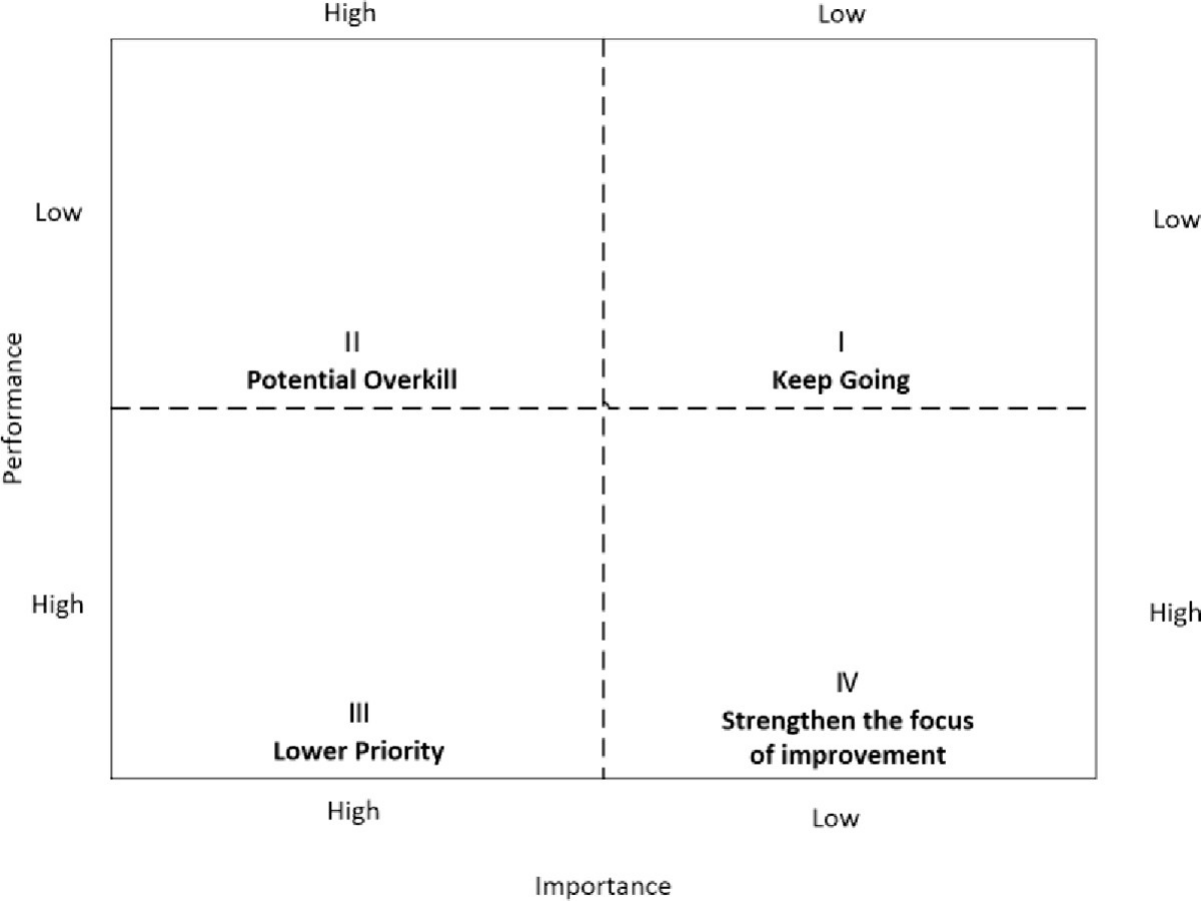
Impact-performance analysis.

IPA, also known as “Action Grid Analysis [50],” is an adaptation of the expected performance method of Olshavsky and Miller [51]. It is based on the assumption that the positive and negative performances of the attributes of the research object may have linear and symmetric effects on the object itself [52]. Based on their performance and importance (which are in turn derived from customer surveys [53]), these attributes are classified into four categories belonging to different quadrants in the action grid, where each quadrant represents a specific strategy (Fig 3) [54,55]. However, many scholars have confirmed an asymmetric relationship [56–59], which means that the positive and negative performances of attributes have a nonlinear effect on resident satisfaction.

**Fig 4:**
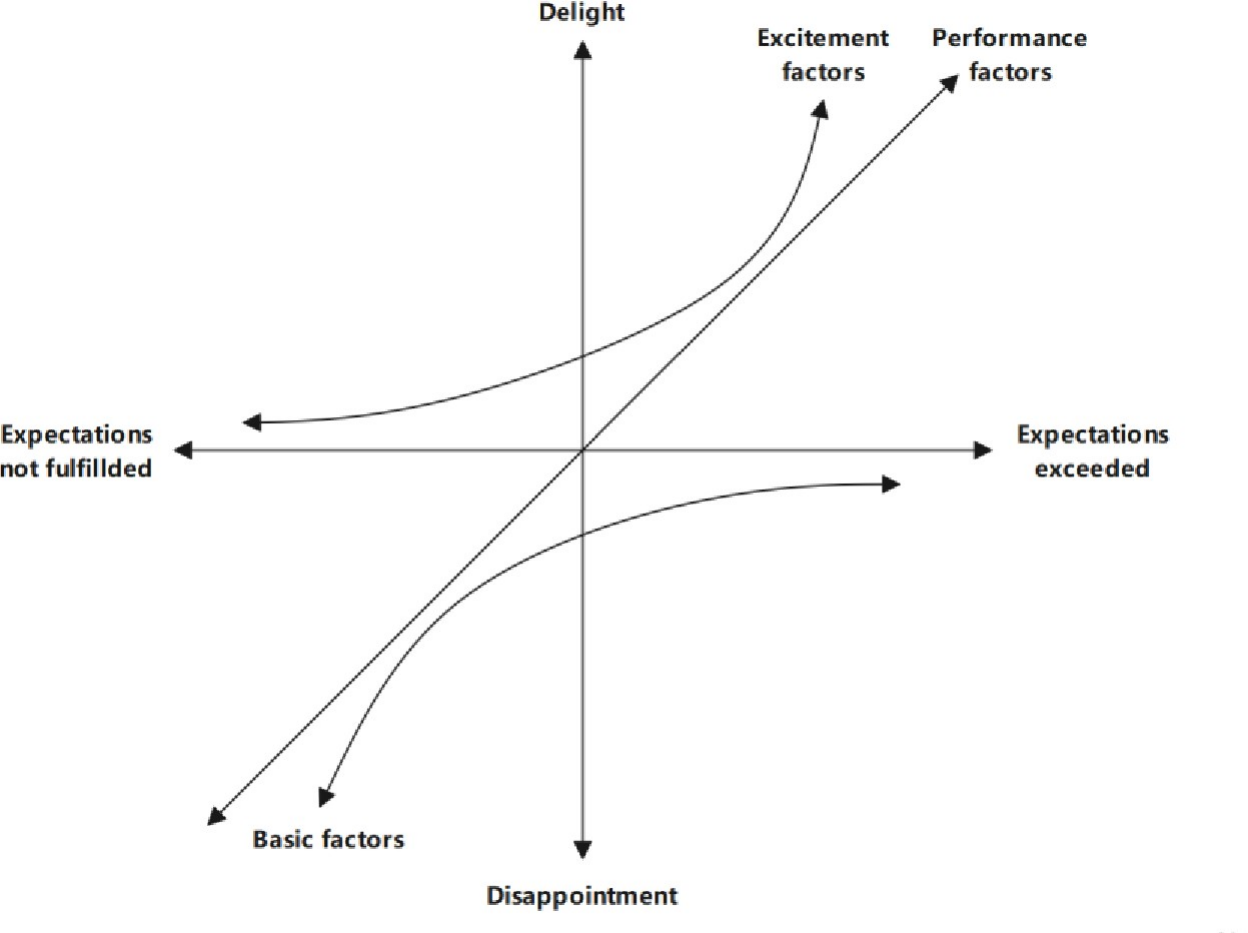
The three-factor theory of customer satisfaction.

Combining the work of Kano et al. [60] with the three-factor customer satisfaction theory, an asymmetric relationship between attributes and satisfaction becomes apparent—satisfaction is not a binary, zero-sum concept; an absence of dissatisfaction does not necessarily indicate satisfaction. According to the nature of the impacts they have on resident satisfaction (which itself is viewed as a spectrum between delight and disappointment) [56,61], attributes are classified into three groups: basic, excitement, and performance factors (Fig 4). The first two attributes have an asymmetric correlation with satisfaction, while the last one has a symmetric relationship. Dissatisfaction rose rapidly when the expectations of basic attributes were not fulfilled; the converse, however, was not true—meeting, or even exceeding [52], these basic expectations only served to decrease disappointment, but did not cause satisfaction or delight. Conversely, meeting or exceeding expectations surrounding excitement factors led to a surge in resident delight, but not fulfilling them only led to a decline in the levels of delight; the sentiment did not cross over to disappointment [62]. Performance factors, on the other hand, have a positive linear relationship with satisfaction, and the level of attribute performance directly determines the extent of satisfaction (delight) or dissatisfaction (disappointment).

Over the years, methods such as the critical incident technique [63,64], importance grid method [65], and penalty-reward contrast analysis (PRCA) [66] have been proposed to quantify asymmetric relations. Among these methods, the third one, which employs dummy-variable regression, is widely used. It encodes customer-perceived performance evaluation data into two groups of dummy variables, one representing higher-level performances with a reward index of *RI* and the other denoting lower-level performances with a penalty index of *PI*. These dummy variables are taken as the independent variable and overall customer satisfaction as the dependent variable for the regression analysis [67].

However, the PRCA criteria for attribute classification are relatively vague. To address this limitation, Matzler and Renzl [68] proposed calculating an impact ratio (*IR*), which is the ratio of the reward index to the absolute value of the penalty index (*IR* = *β*_*ri*_/*β*_*pi*_), and categorizing attributes based on the value of this ratio. When *IR* < 0.8, the attribute is a basic factor; when the ratio is between 0.8 and 1.2, it is a performance factor, while on exceeding 1.2, the attribute becomes an excitement factor. Mikulic and Prebežac [69] proposed, as an extension to PRCA, the impact-asymmetry analysis (*IAA*) method. The impact-asymmetry (*IA*) index is central to this approach and can quantify the categories of the attributes. It is calculated based on reward and punishment indicators and measures the asymmetric impact of attributes on satisfaction by comparing the differences in the degree of satisfaction (or dissatisfaction) contributed by each attribute. IA index values range from -1 to 1; a value close to 1 indicates that the corresponding attribute is an excitement factor, while a value close to -l indicates that the attribute is a basic factor. An index value that is close to neither of the two limits but instead lies between -0.1 and 0.1 is representative of a performance factor.

Caber et al. [70] built on this concept and developed AIPA, a method that uses a two-dimensional matrix composed of the IA index and attribute performance, to display the degree of asymmetric influence of each attribute on overall satisfaction and the performance level of each attribute. This can then be used to propose performance improvement strategies for each attribute. *AIPA* has advantages in visual clarity and intelligibility; its reliability and effectiveness have been verified on comparison with the *IPA* method [62]. This technique has been used in business-to-business and tourism research to identify improvement priorities [62,71].

In summary, the three-factor theory, PRCA, and AIPA, have been widely applied in many research fields, but RORD has not been applied as much. Therefore, this study attempts to:

1. explore the index factors that affect RORD, from the perspective of resident satisfaction, and
2. introduce the three-factor theory, and the AIPA method to explore and quantify the asymmetric relationship between the attributes of old residential districts and residents’ satisfaction, and to determine which attributes must be given priority.

### 2.3 Survey method

**Fig 5:**
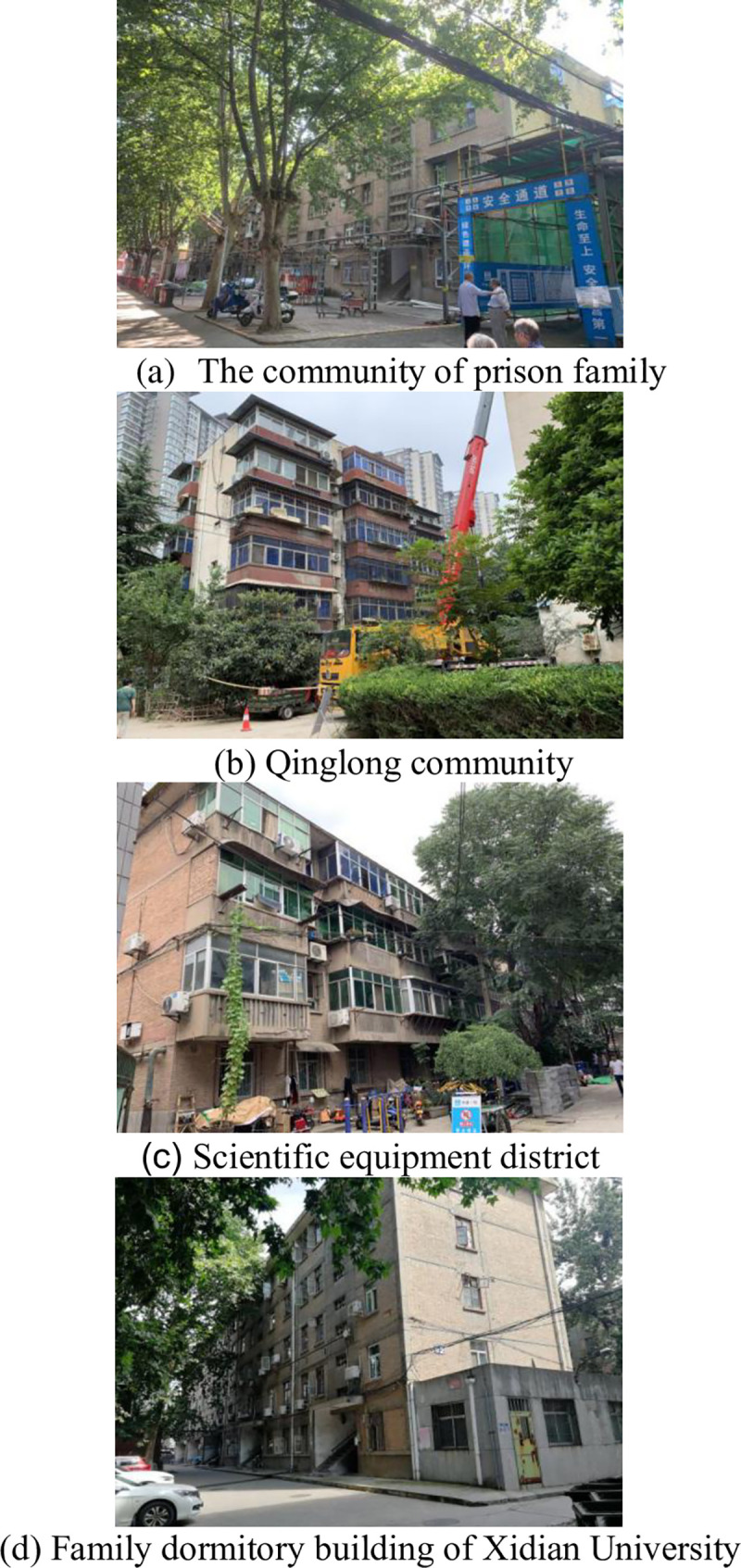
Investigate the status of communities. (a) The community of prison family. (b) Qinglong community. (c) Scientific equipment district. (d) Family dormitory building of Xidian University.

This study surveyed residents of four long-standing communities (Fig 5) in the Yanta and Beilin districts of Xi’an, which is a major city in western China and a prominent national center for scientific research, education, and industrial activities. Located in the middle of the Guanzhong Plain, Xi’an was designated by the UNESCO as a world heritage city in 1981 [72]. It forms the starting point of the Silk Road, is one of the important birthplaces of the Chinese civilization and nation. In February 2018, MOHURD issued a plan to support the transformation of Xi’an into a bustling metropolis with an international transportation hub, while retaining its historical and cultural characteristics.

The responses of residents were recorded in one-to-one questionnaires from September to November 2019 through offline and online methods, the latter involving scanning a QR code with a mobile phone. A total of 380 questionnaires were collected and screened, of which 327 were found to be valid, making for an effective rate of 86.1%. Software programs such as *IBM SPSS Statistics 26* and *Amos Graphics 24* were used to analyze the survey data.

The questionnaire consisted of three parts: attributes of the old residential districts, the overall satisfaction of residents, and the necessary information of respondents. We used the 5-point Likert scale to evaluate the relevance of each attribute, from one denoting zero importance to five denoting the most importance.

**Fig 6:**
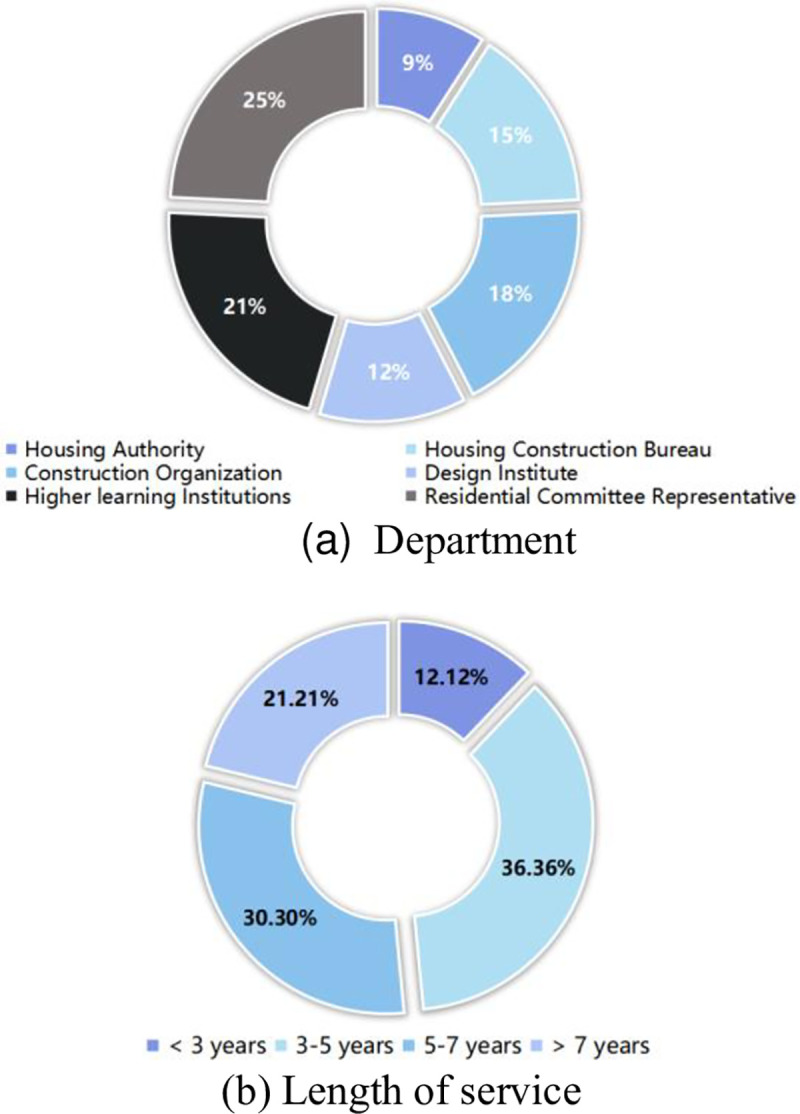
The personnel of the pre-survey. (a) Department. (b) Length of service.

**Fig 7:**
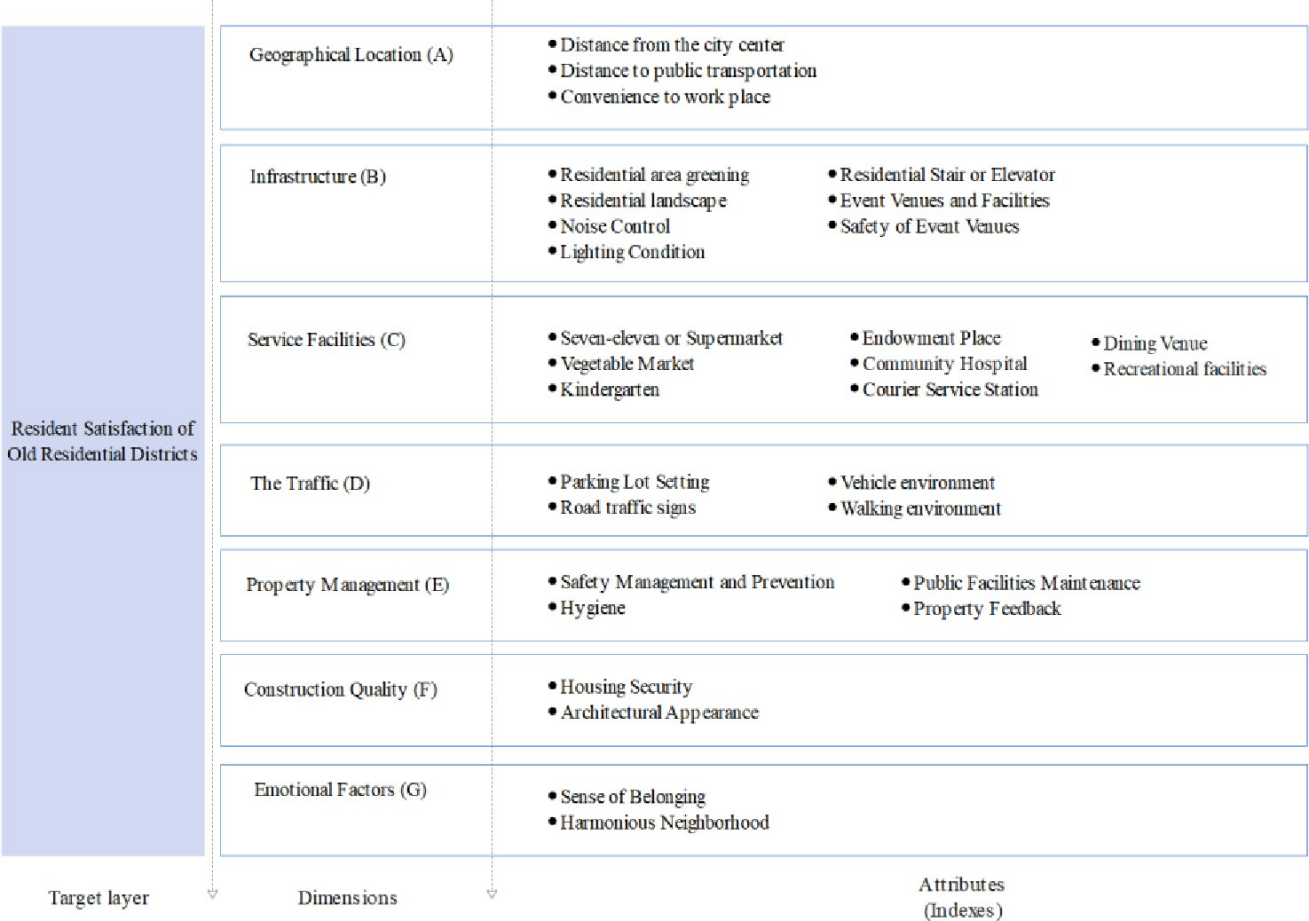
Index system for the renovation of old residential districts.

A pre-survey questionnaire was first distributed to 33 relevant professionals from the housing authority, the housing construction bureau, the construction organization, the design institute, institutes of higher learning, and the residential committee (Fig 6) for testing; they helped optimize the indicator system and the survey questionnaire. Questions were grouped based on index dimensions; the initial index system was derived from problems discovered during earlier investigations, while the final index system with 30 optimized attributes across seven dimensions were derived from the pre-survey analysis and is shown in Fig 7.

## 3. Results

### 3.1 Sample characteristics

The 327 respondents had more females (64.05%) than males (35.95%). Most interviewees (71.17%) were aged between 18 and 35 years, with a good educational background (33.33% had at least one master’s degree or above). Several respondents (22.52%) had a monthly household income of 4001–6000 RMB. Based on the number of years spent residing in their communities, they were nearly evenly divided into three groups: less than 5 years (29.28%), 5–10 years (25.68%), and 11–20 years (26.58%), as shown in Table 1. Although this sample is not entirely representative, the relationships among the research variables (resident perception and satisfaction) captured by this model can be generalized [52,73].

**Table 1:** Sample profile.

Items	Categories	Percentage
Gender	Female	64.05%
	Male	35.95%
Age	18–25	71.17%
	36–60	22.97%
	Over 61	5.86%
Education	Middle school and below	0.45%
	High school	10.36%
	Junior college	19.37%
	Bachelor degree	36.49%
	Graduate degree	33.33%
Income	Under 2000	15.77%
	2001–4000	19.37%
	4001–6000	22.52%
	6001–8000	18.02%
	8001–10,000	12.61%
	Over 10000	11.71%
Length of Residence	Below 5 years	29.28%
	5–10 years	25.68%
	11–20 years	26.58%
	Over 20 years	18.46%

### 3.2 Exploratory factor analysis

Before conducting the exploratory factor analysis, the reliability and validity of the sample data were tested. Reliability analysis showed excellent internal consistency among the test items, with a Cronbach’s alpha value of 0.943 [74]. Validity analysis showed a KMO value of 0.903, and the Bartlett test of sphericity resulted in a chi-square value of 3249.949 (df = 435, p = 0.000). This indicates that the sample data are suitable for factor analysis [75].

Principal component analysis was performed on the exploratory factors. According to Kaiser, factors with eigen values greater than one were extracted first. Items having multiple loads and a factor loading less than 0.5 were deleted by means of the optimal skew rotation method. After three rounds of factor analysis, four attributes—sense of belonging, harmonious neighborhood, endowment place, and community hospital—were eliminated, leaving 26 items to be retained.

These items were then divided into five dimensions with a combined cumulative variance contribution rate of 68.579%, indicating that the five factors account for 68.579% of the information in the scale; this meets the standard minimum requirement of 60% [76]. Results showed the extracted factors to be acceptable (Table 2) and consistent with those of the pre-survey.

- Factor 1 is infrastructure (A), including the attributes of residential area greening (A1), residential landscape (A2), noise control (A3), lighting conditions (A4), residential stairs or elevators (A5), event venues and facilities (A6), and safety of event venues (A7).
- Factor 2 is residential management (B). This includes the combined attribute of property management and construction quality, indicating safety management and prevention (B1), hygiene (B2), public facility maintenance (B3), property feedback (B4), housing security (B5), and architectural appearance (B6).
- Factor 3 is living facilities (C) and constitutes attributes pertaining to service facilities such as dining venues (C1), vegetable markets (C2), courier service stations (C3), 7-Eleven stores or supermarkets (C4), recreational facilities (C5), and kindergarten (C6).
- Factor 4 is traffic (D), including parking lot setting (D1), road traffic signs (D2), vehicle environment (D3), and walking environment (D4).
- Factor 5 is the geographical location (E), including the distance from the city center (E1), accessibility of public transportation (E2), and the distance from workplace (E3).

**Table 2:** The results of exploratory factor analysis.

Dimension	Items	Rotated factors (optimal skew)				
		1	2	3	4	5
Factor 1: Infrastructure	A1	.896				
	A4	.783				
	A2	.743				
	A3	.733				
	A6	.729				
	A7	.622				
	A5	.603				
Factor 2: Residential management	B6		.916			
	B5		.862			
	B2		.854			
	B4		.715			
	B3		.652			
	B1		.553			
Factor 3: Living facilities	C1			.974		
	C5			.717		
	C4			.716		
	C3			.711		
	C2			.700		
	C6			.475		
Factor 4: The traffic	D1				.888	
	D4				.849	
	D2				.727	
	D5				.655	
Factor 5: Geographical location	E1					.881
	E3					.795
	E2					.727
	% of variance	40.180	13.621	5.933	5.020	3.824
	Cumulative%	40.180	53.801	59.734	64.754	68.579
	Standardized Cronbach’s α	0.898	0.908	0.866	0.888	0.804

### 3.3 Confirmatory factor analysis

**Fig 8:**
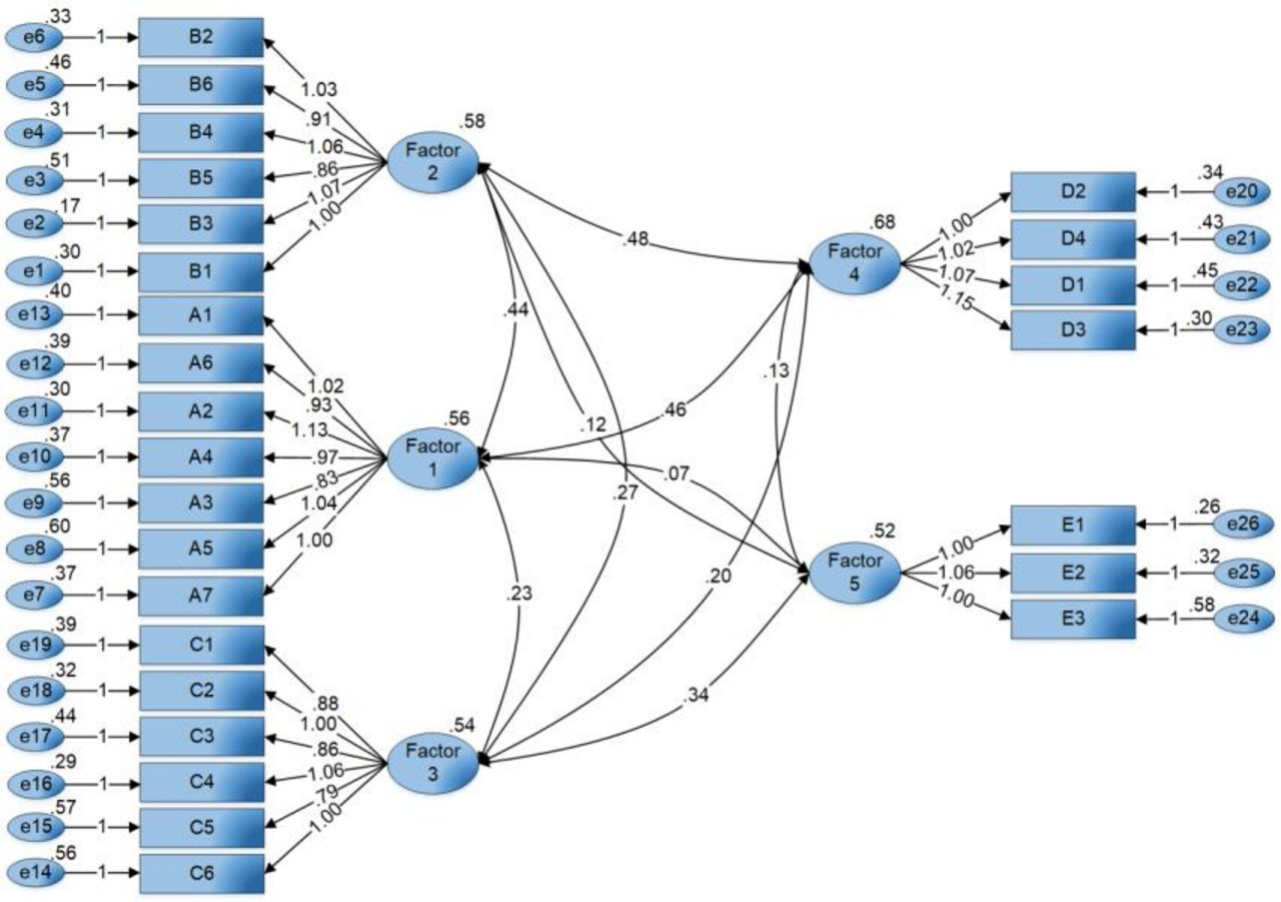
The verification measurement model.

*Amos Graphics 24* was used to test the structural stability of the five factors obtained from the exploratory factor analysis in the previous section. A first-order verification measurement model was constructed by taking the 26 test items as observation variables and the five factors as latent variables (Fig 8), while the maximum likelihood estimation method was used to obtain the relevant adaptation index. As shown in Table 3, all path coefficients reached significantly high values. The X^2^/df ratio was less than 2; several model-fit parameters, such as the comparative fit index(CFI), the normed fit index(NFI), the Tucker-Lewis index(TLI), and the incremental fit index(IFI), were evaluated, and all were found to be between 0 to 1. The root mean square error of approximation(RMSEA) was calculated to be 0.05, indicating a good fit.

**Table 3:** The fitting index of CFA of old residential districts.

Fitting indexes	X2	df	X2/df	RMSEA	CFI	NFI	TLI	IFI
**Model**	**571.393**	**289**	**1.977**	**0.078**	**0.895**	**0.810**	**0.882**	**0.896**

The reliability and validity of each dimension were further tested based on the verification of the measurement model (Table 4). Cronbach’s alpha coefficients for all five dimensions were above 0.8, indicating high reliability. The factor loading of all attributes reached a significant value (between 0.50 and 0.95), denoting that the model’s basic fitness was good [75]. The composite reliability(CR) of the five dimensions exceeded 0.7, while the average variance extracted (AVE) was greater than 0.5, both indicating high aggregation validity for the model. Additionally, the AVE square root of each dimension was greater than the correlation coefficient between them, indicating strong discriminant validity for the model (Table 5).

**Table 4:** The results of confirmatory factor analysis.

No.	Items	Dimensions	Estimate	Cronbach’ s α	CR	AVE
1	A1	Infrastructure	0.770	0.898	0.900	0.563
2	A2		0.838			
3	A3		0.632			
4	A4		0.766			
5	A5		0.711			
6	A6		0.744			
7	A7		0.774			
8	B1	Residential management	0.812	0.908	0.908	0.625
9	B2		0.806			
10	B3		0.893			
11	B4		0.825			
12	B5		0.675			
13	B6		0.713			
14	C1	Living facilities	0.719	0.866	0.868	0.526
15	C2		0.794			
16	C3		0.690			
17	C4		0.821			
18	C5		0.609			
19	C6		0.699			
20	D1	Traffic	0.796	0.888	0.890	0.669
21	D2		0.816			
22	D3		0.868			
23	D4		0.790			
24	E1	Geographical location	0.817	0.804	0.814	0.595
25	E2		0.803			
26	E3		0.687			

**Table 5:** The discriminant validity test results of the measurement model.

	Factor 2	Factor 1	Factor 3	Factor 4	Factor 5
Factor 2	0.791				
Factor 1	0.698**	0.750			
Factor 3	0.458**	0.416**	0.725		
Factor 4	0.684**	0.660**	0.317**	0.818	
Factor 5	0.207**	0.141	0.551**	0.216**	0.771

Note: The diagonal is the square root of AVE.

** p< 0.01, the correlation was significant.

### 3.4 Asymmetric impact-performance analysis

In this study, multiple linear regression analysis was used to determine the asymmetric impact of attributes on resident satisfaction [52]. It is based on dummy-variable regression, which explains how the influence exerted by an attribute on overall satisfaction varies with its performance levels [67]. Before conducting a formal analysis, it is necessary to code each attribute using the corresponding dummy variables. This was done by first dividing the factor scores into quartiles—high, medium, and low. Two dummy variables at the interquartile range were taken as reference points. The first dummy variable was used to measure the impact of higher attribute performance on overall resident satisfaction; attributes with factor scores on or beyond the third quartile were coded as 1, while others were coded as 0. The second dummy variable was used to measure the impact of lower-level performance; attributes with factor scores on or below the first quartile were coded as 1, while others were coded as 0.

Finally, on taking the dummy variable corresponding to attributes of the old residential district itself as the independent variable and the overall satisfaction of residents as the dependent variable, the dummy-variable regression model for the old residential district is constructed as follows [77]:

$$OS_{ORD}=\beta_{0}+\overset{n}{\underset{i=1}{\sum }}\left(\beta_{ri}d_{ri}+\beta_{pi}d_{pi}\right)+\varepsilon$$ (1)

In this formula, *OS*_*ORD*_ denotes the overall satisfaction of residents, *β*_0_ is a constant term, and *d*_*ri*_ and *d*_*pi*_ indicate the high- and low-performing dummy variables of the *i*^*th*^ attribute, while *β*_*ri*_ and *β*_*pi*_ represent the impacts of the higher and lower performances of the *i*^*th*^ attribute on overall satisfaction; *ε* is the residual.

The results showed significant differences between the performance levels (Table 6). Although the values are different at the same performance level, the impact is negative for low-performing attributes and positive for the high-performing ones.

**Table 6:** Asymmetric impact of attributes’ performance.

Attributes	Multiple linear	Regression coefficients of dummy variables		IR
	Regression coefficients	Low performance (*β*_*pi*_)	High performance (*β*_*ri*_)	
Infrastructure	0.063*	-0.840***	0.745***	0.965
Residential management	0.061*	-0.536***	0.672***	1.254
Living facilities	0.062*	-0.622 (n.s.)	0.600**	0.652
The traffic	0.019**	-0.750***	0.768***	0.887
Geographical location	0.073*	-0.699***	0.456***	1.024

Note

* p < 0.1

** p < 0.05

*** p < 0.01; n.s. means insignificant.

Table 6 shows that the low performance of the ‘living facilities’ factor is not significant. However, the high and low performances of other attributes are significant; therefore, *IR* is used to categorize them. When *IR* < 0.8, the attribute is a basic factor; it becomes a performance factor when the ratio is between 0.8 and 1.2, and an excitement factor when it exceeds 1.2. Therefore, residential management is an excitement factor and living facilities are a basic factor, while the other three attributes are performance factors.

AIPA is conducted according to the high and low performance of each attribute [77]. Therefore, the IAA correlation formulas:

$$SGP_{i}=\beta_{ri}/RIOS_{i}$$ (2)

$${\mathrm{DGP}}_{i}=\left|\beta_{pi}\right|/{\mathrm{RIOS}}_{i}$$ (3)

are used to first obtain the IA index of the *i*^*th*^ attribute:

$$IA_{i}=SGP_{i}-DGP_{i},$$ (4)

where *IA*_*i*_ represents the potential of the *i*^*th*^ attribute to generate satisfaction (*SGP*_*i*_) relative to its potential to generate dissatisfaction (*DGP*_*i*_). It is also a quantification of the degree of the asymmetric effect of attribute performance on resident satisfaction. *β*_*ri*_ and *β*_*pi*_ are the high and low performance of the *i*^*th*^ attribute, respectively.*RIOS*_*i*_ = *β*_*ri*_+|*β*_*pi*_| represents the extent to which attribute performance affects overall satisfaction, and *SGP*_*i*_+*DGP*_*i*_ = 1. The results of applying these calculations to all attributes and factors are presented in Table 7.

**Table 7:** Attributes’ performance means and asymmetric impact indexes.

	Means	RIOS	SGP	DGP	Difference ^a^	IA	Category
Infrastructure	18.39	1.222	0.491	0.509	2.78	-0.018	Performance
Residential management	16.95	1.208	0.556	0.444	1.34	0.113	Excitement
Living facilities	20.77	1.155	0.395	0.605	5.16	-0.210	Basic
The traffic	10.57	1.585	0.470	0.530	-5.04	-0.060	Performance
Geographical location	11.38	1.518	0.506	0.494	-4.23	0.012	Performance

Note: “a” represents the difference between the performance score of each attribute and the total average score of 15.61.

The IA index is also a useful indicator of an attribute’s category. When, the attribute is a performance factor; when or, the attribute is an excitement or a basic factor, respectively. This classification of all attributes based on the IA index is shown in the last column of Table 7, which is consistent with the division based on *IR* in Table 6. This mutual corroboration of the two results shows the stability and reliability of the datapoints.

**Fig 9:**
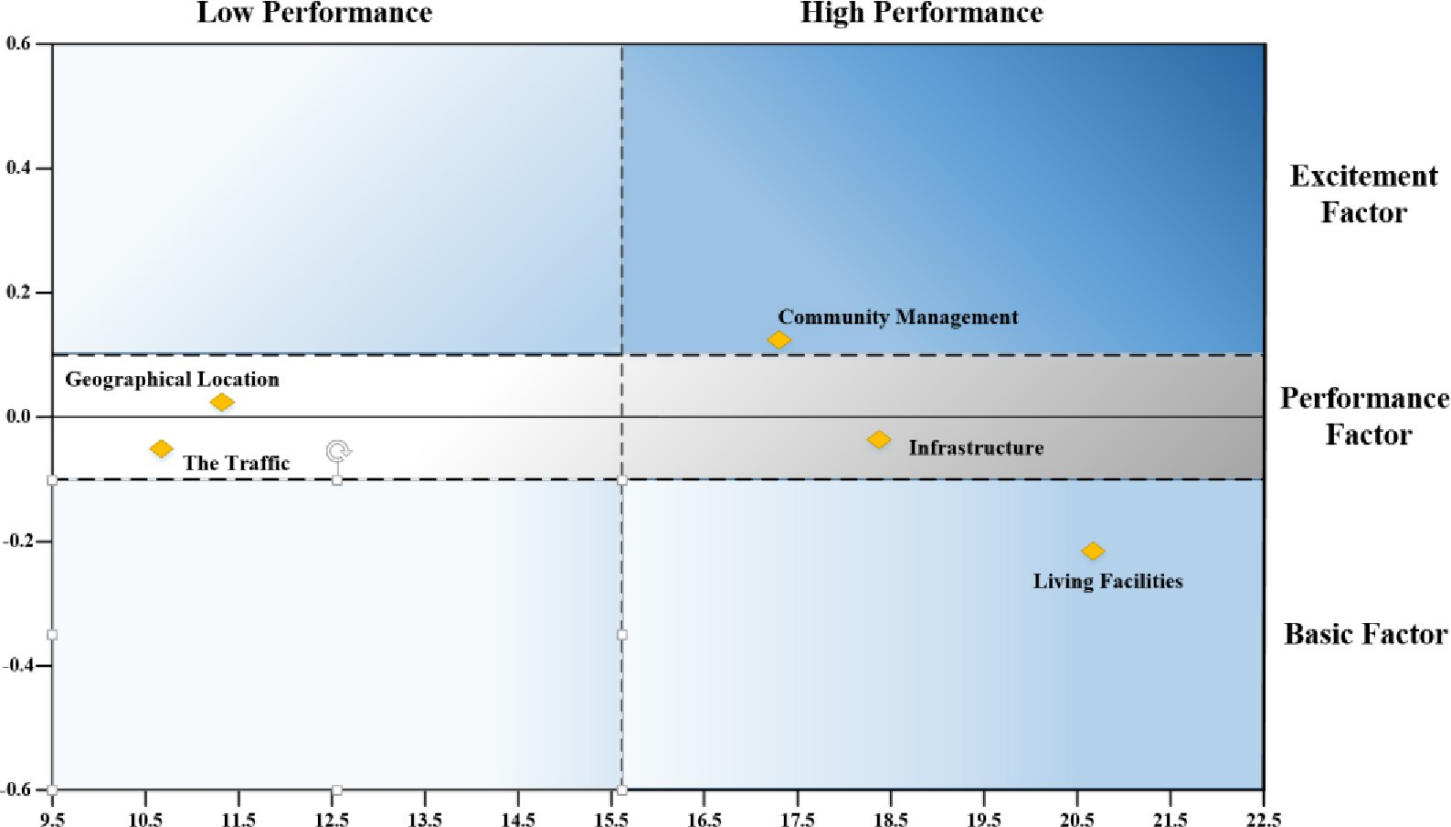
Results on the *AIPA* matrix.

The attribute performance score, calculated as the mean of the items in each dimension, was taken on the horizontal axis and the IA index was plotted along the vertical axis to form a two-dimensional matrix for the AIPA computation. Crossing the vertical axis to make two horizontal auxiliary lines at *IA* = −0.1 and *IA* = 0 showed the three-factor classification of attributes. A vertical auxiliary line at a performance score of 15.61 was made across the horizontal axis to highlight the performance level of the attributes (Fig 9). This matrix can be used to determine the required RORD strategies based on attributes, asymmetric impact types, and performance.

## 4. Discussion

China’s urban construction has begun to shift from incremental planning to inventory planning. Increasing demands of residents for better living environments need to be balanced with the prevailing strict restrictions on land index. Given the shortage of construction land resources, economic utilization of existing ones is the need of the hour [78,79]. Since old residential districts form the largest chunk of urban construction land, their renovation is likely to become a development trend. Additionally, residential communities in large-scale constructions are likely to enter the ranks of old residential districts with time. Therefore, it is necessary to meet the spatial demand of future urban development with the renewal of inventory land and the demands of residents with RORD, which is an essential part of urban inventory planning.

Prioritizing the transformation content is one of the key steps in the regeneration and utilization of old residential areas. It can effectively utilize and develop urban stock land, and assist planners and managers in determining the priority between resources and attributes. It can also improve the living environment and quality of living in old communities.

In this study, AIPA was chosen for classification of the attributes of the old cell and propose a transformation strategy. This is an effective technique based on the asymmetric impact of attributes on overall satisfaction and performance levels.

Results show residential management, infrastructure, traffic, geographical location, and living facilities to be the most influential dimensions of resident satisfaction in these districts; these are the attributes that the government should pay more attention to during renovation.

### 4.1 Dimension 1: Living facilities (C1–C6)

AIPA results show that living facilities are an essential high-performance factor. Since basic factors affect satisfaction negatively and asymmetrically (i.e., the basic factors exceeding the prescribed requirements has no appreciable impact on satisfaction, but not meeting the prescribed requirements causes significant dissatisfaction), the current status of living facilities should be preserved, while its performance must be maintained above a certain level. Moreover, the IA index of living facilities is -0.210, which also indicates that residents are highly likely to be dissatisfied by it. The factor loading in Table 4 shows the values of C1–C4 and C6–C7 to be relatively high, indicating that the commercial facilities around the settlements can satisfy the daily needs of residents. The value of recreational facilities (C5) is low (0.609), recommending the enrichment of leisure and activity places around the community, so that people can have their entertainment needs met nearer to home during the holidays.

### 4.2 Dimension 2: Residential management (B1–B6)

The AIPA matrix also shows that residential management is an excitement factor with high performance, which means that maintaining current levels of performance is good enough to generate satisfaction. Excitement factors have a positive asymmetric effect on resident satisfaction—when its performance is high, its effect on the degree of satisfaction conforms to the law of increasing marginal revenue. Because the performance of residential management (16.95) is only slightly higher than the average value (15.61), effective measures should be taken to improve lower-weight attributes in order to maintain the current status. More specifically, it can be seen from Table 4 that the six attributes of residential management have higher factor loadings. Among them, those of housing security (B5) and architectural appearance (B6) are relatively low (0.675 and 0.713, respectively). Therefore, effective promotional strategies should be adopted. Due to the age of the buildings in these districts, not only does their façade need to be restored, but also the internal facilities need to be repaired. These include the unit entrance, the heating system, wall insulation, building sanitation, wall beautification, etc.

### 4.3 Dimension 3–5: Traffic, geographical, and infrastructures (D1–D4, E1–E3, and A1–A7)

**Fig 10:**
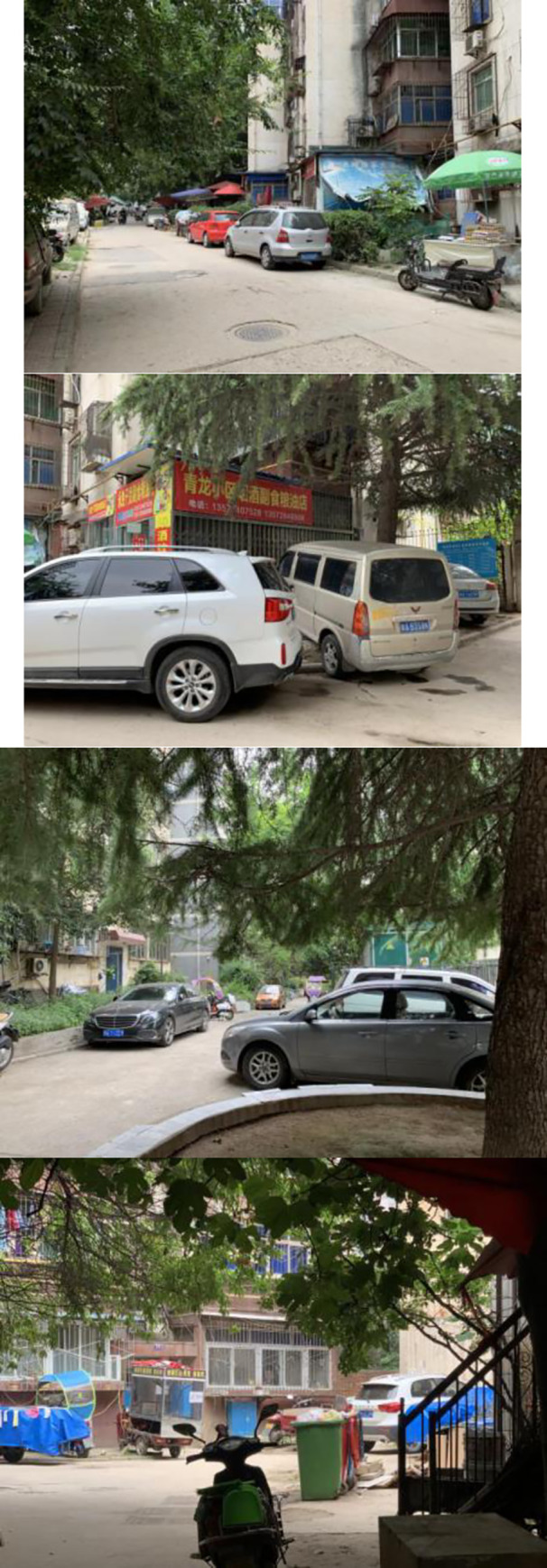
The phenomenon of random parking.

Unlike the first two attributes, traffic, geographical location, and infrastructures were all performance factors. Performance factors affect overall resident satisfaction linearly and symmetrically—i.e., any increase or decrease in their performance will lead to similar and corresponding changes in satisfaction. Hence, these three dimensions must maintain high levels of performance to ensure resident satisfaction; sub-par performance of these attributes is not acceptable. Among them, traffic and geographical location were found to be low-performance factors—their performance is far below average, dissatisfying residents. Regarding geographical location, most residences were located at the heart of the city when they were constructed. Rapid urbanization of surrounding areas with time, however, caused the central area of the city to gradually shift elsewhere, leaving the residential locality in an area that is currently marginalized. Newer areas are developing faster with increasingly well-connected networks of business and transportation. Therefore, the government should also consider this factor during the renovation process—shared public vehicles such as bikes and electric vehicles can be set up between the old districts and the surrounding newer areas to alleviate the transportation inconvenience experienced by residents. Traffic had the lowest performance among all dimensions (10.57). According to the survey, the prevailing state of parking and traffic conditions significantly reduced living satisfaction (Fig 10). The parking of motor and non-motor vehicles in the community is disorderly, happening at will and often occupying the road. The lack of parking spaces and the encroachment of parked vehicles on pedestrian passageways, results in the frequent mixing of traffic between people and vehicles. This poses several safety hazards and severely affects the landscape of the area and the daily life of residents, leading to a significant reduction in their satisfaction. Communities built at an earlier time have a larger area; some of them are in a district state, with more buildings and irregular distribution. Signs guiding people to the buildings, parking facilities, and through prevailing traffic conditions in the locality should be set up to facilitate the movement of people. Thus, the parking problem was found to be of two types: abundant and narrow internal spaces; effective measures should be taken to alleviate them.

Infrastructure is a high-performance factor (with an IA index of -0.018). This shows that the infrastructure should not only maintain its current high performance, but also take effective targeted measures to avoid resident dissatisfaction. Specifically, it can be seen from Table 4 that the factor loadings of noise control (A3) and residential stairs/elevator (A5) are low (0.632 and 0.711, respectively). Therefore, it would be prudent for the government to attach much importance to noise control during renovation; erecting no-honking signs around these districts could be one way of doing it. Elderly residents account for a large proportion of the population of these districts; residences being multistoried buildings without elevators makes it inconvenient for those living on higher floors to take the stairs every day. Installing elevators is a time-consuming, costly, and labor-intensive project. The cost remaining after the government subsidy can be shared by house owners on higher floors (i.e., excluding the first and second floors). Therefore, considering the rights and interests of all households, the installation of elevators is a much-needed solution.

According to the AIPA technology, the priority of the content of the urban old residential district renovation can be determined. The methods and strategies for improving the environment of the old residential district have been proposed, keeping the residents’ feelings at the core. At the same time, it is also necessary to discuss the limitations and precautions of the interpretation results. This study considers the characteristics of residents’ life, the built environment, and road traffic. However, it does not include the characteristics of other dimensions that also affect residents’ satisfaction with existing settlements, such as the characteristics of society and economy. A more comprehensive outlook on excluded dimensions would be included in our future research. Additionally, the attributes and localities employed in this study are limited to a single city–it does not allow comparison across different cities; as a result of which, it is unable to account for the corresponding deviations in the attributes when compared across different cities. While the AIPA method quantifies the nonlinear impact of some attributes on resident satisfaction, the reason behind it has not been explained. This provides scope for further research, where complex functions can be introduced to explore the mechanism behind the impact of these attributes on resident satisfaction.

## 5. Conclusion

This research proposes a collaborative method to determine the priorities of the old residential district renovation that involves community participation, through introduction of AIPA technology into the field of sustainable urban development. In general, the renovation of old residential districts plays an important role in refinement of sustainability and living conditions of urban development. Community participation and residents’ satisfaction regarding the renovation, must be prioritized in the process of urban development and construction. This research provides a framework for the renovation of urban old residential districts, based on resident satisfaction. The first step involved clarification of the residents’ perception of the attributes of the old residential districts, and their satisfaction regarding the renovation. Next, we computed significant predictors of overall satisfaction. Further, we identified the asymmetric impact of attribute performance on overall satisfaction, and divided the attributes into three groups. Finally, the attributes were positioned on the AIPA matrix, by integrating the three factors and attribute performance. An improvement strategy for the renovation of old residential districts has been proposed on the given basis, and the priority of the renovation content was determined.

Based on the three-factor theory of customer satisfaction and its empirical research method, this study considers four representative localities in the Yanta and Beilin districts of Xi’an City in China as a case study to further explore the application of asymmetric influence relationships in the study of resident satisfaction. In this study, the attribute dimensions of old residential districts were investigated using factor analysis. Then, the asymmetric influence of the attribute performance of old residential districts on the overall satisfaction of residents was analyzed by PRCA and AIPA. Results showed that,

1. the attributes of old residential districts can be classified into five dimensions: residential management, infrastructure, traffic, geographical location, and living facilities;
2. the performance of these attributes has an asymmetric impact on the overall satisfaction of residents. Living facilities, which are a basic factor, have a negative asymmetric effect on satisfaction, while residential management, an excitement factor, has a positive asymmetric effect on satisfaction. The influence of performance factors such as infrastructure, traffic, and geographical location on satisfaction is symmetrical. However, the influence of emotional factors on residents’ satisfaction was not found to be significant.
